# Impact of severe acute respiratory coronavirus virus 2 (SARS-CoV-2) vaccination on the incidence of coronavirus disease 2019 (COVID-19) among healthcare workers at an academic medical center in Vermont

**DOI:** 10.1017/ash.2022.15

**Published:** 2022-02-28

**Authors:** Cindy D. Noyes, Monica J. Raymond, Christina M. Wojewoda, W. Kemper Alston

**Affiliations:** 1 Department of Medicine, University of Vermont Medical Center, Burlington, Vermont; 2 Infection Prevention, University of Vermont Medical Center, Burlington, Vermont; 3 Pathology and Laboratory Medicine, University of Vermont Medical Center, Burlington, Vermont

## Abstract

Infection prevention strategies and vaccination reduce risk of severe acute respiratory coronavirus virus 2 (SARS-CoV-2) transmission to healthcare workers (HCWs). We describe coronavirus disease 2019 (COVID-19) incidence and vaccination rates in a cohort of HCWs at the University of Vermont Medical Center. Before vaccines, the HCW COVID-19 incidence paralleled that of the State of Vermont; after vaccination, incidence fell and remained low.

Healthcare workers (HCWs) have risk for occupational exposure to severe acute respiratory coronavirus virus 2 (SARS-CoV-2), the virus that causes coronavirus disease 2019 (COVID-19).^1,2^ Personal protective equipment (PPE), engineering controls, physical distancing, detection, and restriction of infected HCWs reduce the risk of nosocomial infection. When PPE is available and infection prevention strategies are utilized, the risk for COVID-19 has been associated with rates of illness from the community rather than occupational exposure.^3,4^ Early in the pandemic, the University of Vermont Medical Center (UVMMC) prioritized PPE for personnel with greatest risk of COVID-19 exposure. Universal masking was initiated for all staff, patients, and visitors as well as universal eye protection for staff during inpatient encounters. HCWs caring for patients with confirmed or suspected COVID-19 wore gowns, gloves, N95 or other approved respirators, and eye protection. All HCWs wore respirators and eye protection during aerosol-generating procedures. Visitation was restricted. In December 2020, the first COVID-19 vaccine received emergency use authorization, and UVMMC immediately began vaccinating employees. In this study, we sought to determine whether HCWs at UVMMC were at higher risk of COVID-19 than Vermont residents in general. We also examined the impact of rapid vaccine uptake in HCWs, and we have described the impact of vaccination on polymerase chain reaction (PCR) cycle threshold (Ct).

## Methods

This observational study of HCWs was conducted at UVMMC, a 500-bed, academic medical center in Burlington, Vermont. It is the teaching hospital for the University of Vermont Larner College of Medicine, and it is a tertiary-care center serving 1 million people in Vermont and New York. All 6,844 employees of UVMMC were considered HCWs for this study. HCW vaccination began December 15, 2020 for patient-facing HCWs. HCWs without patient contact or who worked off site were not vaccinated until they met state eligibility. The study period was chosen as October 4, 2020, to May 1, 2021, because this period included the highest number of cases as well as vaccine rollout for HCWs. HCWs were instructed to call a hotline with symptoms, exposure, quarantine due to travel, or other concerns. Employee health department staff performed symptom assessment and exposure risk evaluation, and they facilitated testing and restriction of HCWs. HCWs were considered cases if they had a positive SARS-CoV-2 PCR or antigen test combined with either COVID-19 symptoms or an epidemiologic link to a confirmed case. Infection prevention staff interviewed all cases for contact tracing and source of exposure. All inquiries, evaluation, test results, and vaccinations were confirmed using employee health records and were documented in a shared database. The incidence of HCW infections at UVMMC was compared to the publicly reported incidence for the State of Vermont. The vaccination rate for UVMMC HCWs was compared to the vaccination rate for the State of Vermont. Cycle threshold (Ct) values were obtained for each fully vaccinated case for HCWs and were compared to the Ct values of 4 randomly selected unvaccinated HCW cases. Testing platforms varied based on institutional triage and assay capacity and included Quantstudio 7 (Applied Biosystems, Waltham, MA), Cobas 6800 (Roche Diagnostics, Indianapolis, IN), GeneXpert (Cepheid, Sunnyvale, CA), Panther Fusion (Hologic, Marlborough, MA), and those sent to the Broad Institute (Cambridge, MA). The SARS-CoV-2 PCR Ct value represents the number of amplification cycles needed to detect genetic targets and is inversely proportional to viral burden. The test is not designed as a quantitative assay; however, a lower Ct represents more viral RNA in the sample.^5,6^ Some laboratory platforms report Ct values for 2 targets. Lacking guidance from the literature, the mean was calculated to obtain a single value for each case for analysis. The median Ct values of vaccinated and unvaccinated cases were compared using the Wilcoxon rank-sum test performed in STATA version 15.1 software (StataCorp, College Station, TX). Institutional review board approval was obtained from the University of Vermont.

## Results

**Fig. 1.**
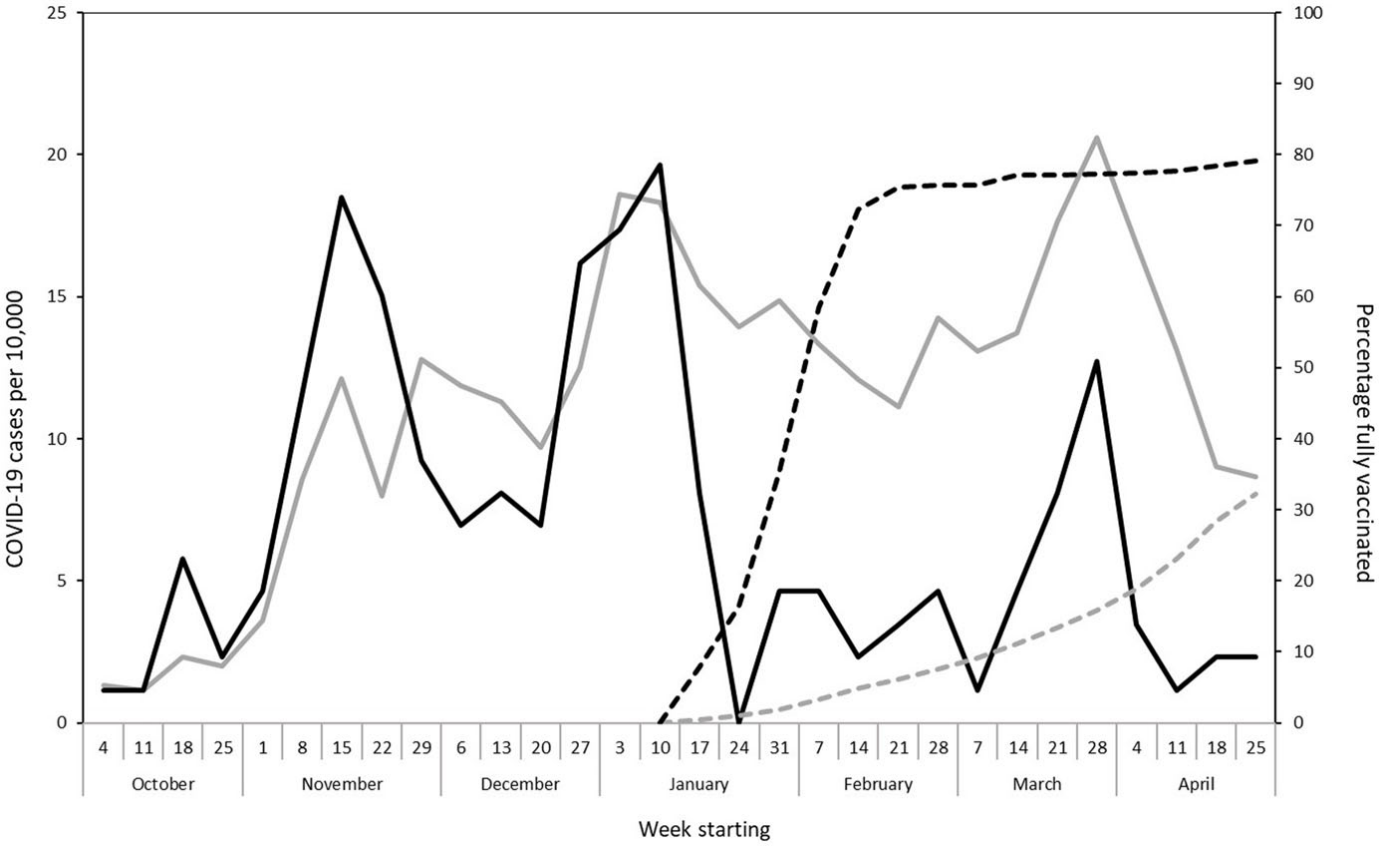
COVID-19 cases per 10,000 by date of specimen collection and percentage fully vaccinated, University of Vermont Medical Center employees versus the state population-Vermont, October 4, 2020–May 2021. 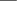 Vermont State cases per 10,000. 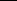 Medical center cases per 10,000. 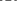 Vermont state % fully vaccinated. 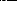 Medical center % fully vaccinated.

**Fig. 2.**
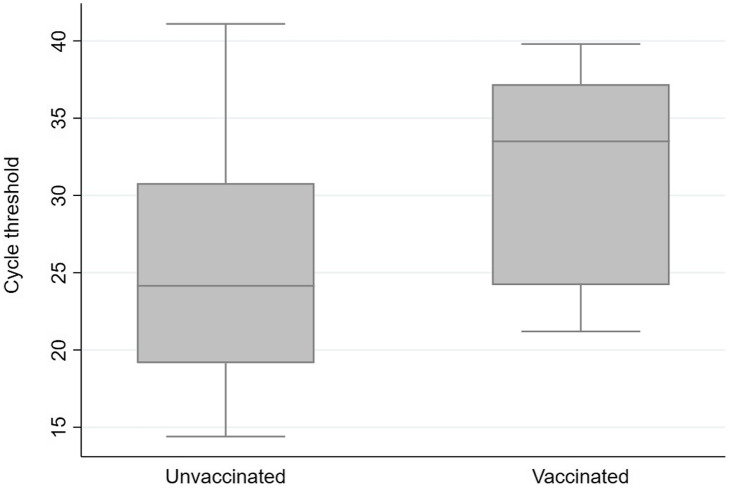
Comparison of positive SARS-CoV-2 PCR cycle-threshold results by vaccination status, University of Vermont Medical Center employees, October 4, 2020, to May 1, 2021. Employees were considered vaccinated if they had received a complete COVID-19 vaccine series at least 14 days prior to illness onset, and they were considered unvaccinated if they had never received a dose of COVID-19 vaccine. Note. SARS-CoV-2, severe acute respiratory syndrome coronavirus 2; PCR, polymerase chain reaction.

By February 27, 2021, 75% of eligible UVMMC HCWs were fully vaccinated (i.e., they were 14 days past a completed vaccine series). From October 4, 2020, to May 1, 2021, 180 HCWs tested positive for COVID-19 for the first time. Among them, 137 were unvaccinated, 30 were partially vaccinated (i.e., had received dose 1 at least 1 day prior to illness onset and within 14 days of dose 2), and 13 were fully vaccinated. Symptoms were reported by 111 (81%) of the 137 unvaccinated cases and 9 (69%) of the 13 fully vaccinated HCWs. The incidence of COVID-19 in UVMMC HCWs mirrored the incidence in the State of Vermont until 4 weeks after HCW vaccination began, when cases among HCWs dramatically decreased and remained low despite persistent community transmission. HCW infections continued to parallel community activity, though at a lower rate (Fig. 1). Of the 110 cases that followed vaccine rollout, 97 cases (88%) developed in those who were unvaccinated or partially vaccinated. Of 6,844 fully vaccinated HCWs, 13 tested positive for SARS-CoV-2 14 or more days after vaccination (range, 16–91 days) for a breakthrough rate of 0.19%. All 13 of these HCWs reported household contact with 1 or more infected persons. The median Ct value was higher in the 13 fully vaccinated HCWs (median Ct, 33.5) than in the 52 randomly selected cases of unvaccinated HCWs (median Ct, 24.2; *P* < .01) (Fig. 2). The median Ct values were lowest in symptomatic, unvaccinated HCWs (median Ct, 21.9) compared to other infected HCWs in the sample (median Ct, 31.4; *P* < .01).

## Discussion

The study was performed from October 2020 to May 2021, prior to detection of the SARS-CoV-2 δ (delta) variant in Vermont. Prior to vaccination, the SARS-CoV-2 infection rate among HCWs at UVMMC was nearly identical to that of the State of Vermont overall, suggesting that infection prevention strategies, such as vigilant PPE use, universal masking, testing and restriction of infected staff and visitor restriction were effective in limiting the transmission of SARS-CoV-2 to HCWs within the institution. Additionally, extensive contact tracing yielded household and family exposures as the most common source of HCW infection. One month after vaccination began, HCW infection rates plummeted and remained low despite persistent COVID-19 transmission in the community. These data support the real-world vaccine efficacy documented by others.^7^ Breakthrough infections for fully vaccinated HCWs were rare (0.19%) and resulted in asymptomatic or mild disease in 12 of 13. Although the Infectious Disease Society of America and the Association of Molecular Pathology urge caution when using PCR Ct values for routine clinical decision making for COVID-19 patients, Ct values have been used as a proxy for infectivity.^8,9^ In our study, the median Ct value for vaccinated cases was higher than that for unvaccinated cases. This difference suggests that SARS-CoV-2 infection following vaccination may result in lower viral loads and a reduced risk of transmission.

This study had several limitations. Multiple PCR platforms were used for testing, which may limit the validity of Ct comparisons. Due to small sample sizes, matching for testing platform for Ct comparison was not performed. Testing was offered but was not mandatory for vaccinated asymptomatic HCWs after high-risk exposures, possibly reducing the number of breakthrough infections identified. Of the vaccinated HCWs who tested positive, 4 were asymptomatic. Viral cultures were not available; thus, it remains unclear whether these positive tests reflected a replication-competent virus. Finally, this single institutional study was conducted in a low-prevalence state, which limits the generalizability of these findings to other facilities or regions.

In conclusion, prior to vaccination, HCWs were not at greater risk of COVID-19 than the general population of Vermont, suggesting that infection prevention strategies were effective in limiting transmission to HCWs in the workplace. UVMMC experienced a precipitous decrease in incidence of infection early in our HCW vaccine program. Breakthrough infections were rare and did not result in hospitalizations or death. The high median Ct values among breakthrough cases raises the possibility that infected, vaccinated persons have lower viral loads than unvaccinated cases.

## References

[1] Nguyen LH , Drew DA , Graham MS , et al. Risk of COVID-19 among frontline healthcare workers and the general community: a prospective cohort study. Lancet Public Health 2020; 5:e475–e483. 32745512 10.1016/S2468-2667(20)30164-X PMC7491202

[2] Kambhampati AK , O’Halloran AC , Whitaker M , et al. COVID-19 hospitalizations among health care personnel—COVID-NET, 13 states, March 1–May 31, 2020. Morb Mortal Wkly Rep 2020;69:1576–1583. 10.15585/mmwr.mm6943e3 PMC7659917 33119554

[3] Jacob JT , Baker JM , Fridkin SM , et al. Risk factors associated with SARS-CoV-2 seropositivity among US healthcare personnel. JAMA Network Open 2021;4(3):e211283. 33688967 10.1001/jamanetworkopen.2021.1283 PMC7948059

[4] Trieu M , Bansal A , Madsen A , et al. SARS-CoV-2–specific neutralizing antibody responses in Norwegian healthcare workers after the first wave of the COVID-19 pandemic: a prospective cohort study. J Infect Dis 2021;223:589–599. 33247924 10.1093/infdis/jiaa737 PMC7798943

[5] Jefferson T , Spencer EA , Brassey J , Heneghan C. Viral cultures for COVID-19 infectious potential assessment—a systematic review. Clin Infect Dis 2021;73:e3884–e3899. 33270107 10.1093/cid/ciaa1764 PMC7799320

[6] Ontario Agency for Health Protection and Promotion. Focus on: an overview of cycle threshold values and their role in SARS-CoV-2 real-time PCR test interpretation. Public Health Ontario website https://www.publichealthontario.ca/-/media/documents/ncov/main/2020/09/cycle-threshold-values-sars-cov2-pcr.pdf?la=en. Accessed August 11, 2021.

[7] Thompson BG , Burgess JL , Naleway AL , et al. Interim estimates of vaccine effectiveness of BNT162b2 and mRNA-1273 COVID-19 vaccines in preventing SARS-CoV-2 infection among healthcare personnel, first responders, and other essential and frontline workers—eight US locations, December 2020–March 2021. Morb Mortal Wkly Rep 2021;70:495–500. 10.15585/mmwr.mm7013e3 PMC8022879 33793460

[8] Infectious Diseases Society of America and Association for Molecular Pathology. IDSA and AMP joint statement on the use of SARS-CoV-2 PCR cycle threshold (Ct) values for clinical decision making, 2021. IDSA website. https://www.idsociety.org/globalassets/idsa/public-health/covid-19/idsa-amp-statement.pdf. Accessed August 11, 2021.

[9] Bullard J , Dust K , Funk D , et al. Predicting infectious SARS-CoV-2 from diagnostic samples. Clin Infect Dis 2020;71:2663–2666. 32442256 10.1093/cid/ciaa638 PMC7314198

